# Association of Self-Perceived Oral Health and Function with Clinically Determined Oral Health Status among Adults Aged 35–54 Years: A Cross-Sectional Study

**DOI:** 10.3390/ijerph15081681

**Published:** 2018-08-07

**Authors:** Se-Yeon Kim, Ji-Eun Kim, Han-Na Kim, Eun-Joo Jun, Jung-Ha Lee, Ji-Soo Kim, Jin-Bom Kim

**Affiliations:** 1 Department of Preventive and Community Dentistry, School of Dentistry, Pusan National University, Yangsan 50612, Korea; secan00@naver.com (S.-Y.K.); jieunee9@hanmail.net (J.-E.K.); jsilver@pusan.ac.kr (E.-J.J.); jhlee86@pusan.ac.kr (J.-H.L.); psily1@naver.com (J.-S.K.); 2 BK21 PLUS Project, School of Dentistry, Pusan National University, Yangsan 50612, Korea; 3 Department of Dental Hygiene, College of Health and Medical Sciences, Cheongju University, Cheongju 28503, Korea; hnkim@cju.ac.kr

**Keywords:** oral function ability, oral health, oral health perceptions, periodontal status, prosthetic status

## Abstract

This study aimed to analyse the association of self-perceived oral health status (OHS) and functions with clinical OHS in Korean adults aged 35–54 years. The study was designed as a cross-sectional study using data from the Fourth Korea National Health and Nutrition Examination Survey (2007–2009). A total of 6605 subjects aged 35–54 years who completed the oral examination and questionnaires were included. An association of self-perceived OHS and functions with clinically determined OHS was confirmed by a complex-samples general linear model. Data on socioeconomic variables, i.e., household income and education level, self-perceived OHS and functions, such as chewing and speaking, were collected by trained interviewers. The clinical OHS was determined by trained dentists and included the number of untreated decayed teeth (DT); decayed, missing, and filled teeth (DMFT); prosthetic and periodontal status. The combined score was estimated as the sum of self-perceived OHS and functions. Based on the estimation coefficient, the clinical variables that were most strongly associated with self-perceived OHS and functions were, in order, periodontal status, prosthetic status, DT, and DMFT. In addition, the combined score for self-perceived OHS and functions was associated with household income, education, and clinically determined OHS.

## 1. Introduction

Oral health is one of the domains of health that can affect general body functions, and hence, the overall sense of well-being. Good oral health is an important component of general health, since chewing ability is an essential function that promotes health and quality of life [1,2]. However, many adults lose teeth due to oral diseases, such as caries and periodontal disease [3,4]. Tooth loss frequently causes some difficulty in masticatory performance and swallowing [5,6]. In particular, many individuals wearing dentures have a poor nutrient intake and are vulnerable to disease. The bite and masticatory forces of denture wearers is reportedly only approximately 20–25% of that of dentate individuals [7], and denture wearers have limited chewing efficiency [8]. 

Previous studies have identified several factors related to self-rated oral health [9,10,11,12,13]. The improvement of oral health literacy has been associated with better oral health status (OHS) [9,10]. However, knowledge is not sufficient to change behaviour; social determinants were associated with a risk of poor oral health [11]. Assessment and understanding of self-rated oral health should take into account social factors, as well as subjective and clinical (objective) oral symptoms [12,13]. 

Objective and subjective methods are used to evaluate oral health. Objective evaluation is conducted by a dentist, while subjective self-rating is dependent on the individual’s own perception of OHS [12]. In some cases, dentists and patients may have different opinions concerning OHS [14,15]. Several studies have addressed the importance of agreement between objective evaluation and subjective OHS, but these studies have been limited to epidemiological variables. In previous studies, the decayed, missing, and filled teeth (DMFT) index or periodontal or prosthesis status have been considered individually, but their relationship with each other has not been assessed [16,17]. Therefore, it is necessary to elucidate the association between objective and subjective OHS in a comprehensive model that includes clinical variables as well as demographic and socioeconomic variables and health-related behaviours.

The aim of this study was to analyse the association of subjective OHS and functions with clinically determined OHS among adults. In terms of the relationship between oral function and dentition, the number of teeth—below a minimum of 20—is associated with impaired masticatory efficiency, performance, and ability [18]. Moreover, individuals with diverse OHS are found across all age groups. Since it is difficult to analyse all age groups, we selected individuals with a similar number of existing teeth and determinable periodontal disease [19,20]. Consequently, this study targeted adults aged 35–54 years with similar dentition. 

## 2. Materials and Methods

### 2.1. Data Sources

This study was based on data obtained from the Fourth Korea National Health and Nutrition Examination Survey (KNHANES-IV), which was conducted from 2007 to 2009 by the Korea Centers for Disease Control and Prevention (KCDCP). KNHANES is conducted annually using a rolling sampling design, which involves a complex, stratified, multistage probability-cluster survey of a large representative sample of the non-institutionalized civilian population in South Korea. The purpose of this survey was to gather national data about the health status, awareness, and behaviour, as well as the nutritional intake of the Korean population. KNHANES-IV included highly structured health-related questionnaires, a nutrition survey, and an oral health examination conducted by trained dentists [21,22]. 

**Figure 1:**
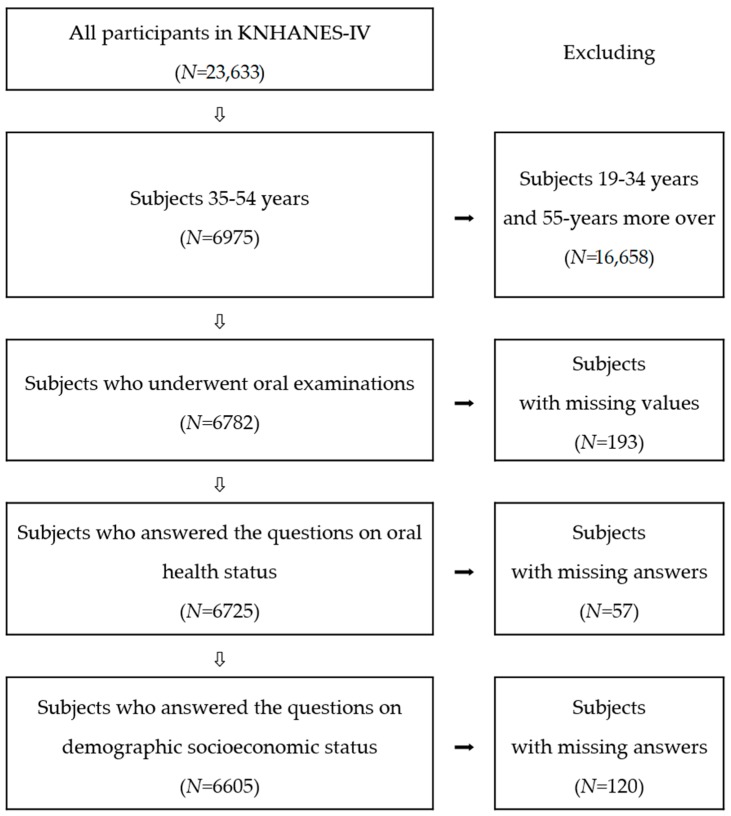
Flow chart for selection of study participants (KNHANES-IV: Fourth Korea National Health and Nutrition Examination Survey).

This cross-sectional analysis was restricted to adult participants aged 35–54 years, among all 23,633 participants in KNHANES-IV, who had undergone the oral health examination and completed the health-related questionnaires (15,633,436 weighted samples). Subjects with one or more missing answers in the questionnaires, and subjects without oral health examination data, were excluded from the analysis. We eventually included 6605 individuals who had undergone clinical oral health examinations and completed the questionnaire, which included demographic socioeconomic variables, self-perceived OHS, and oral functions (chewing and speaking) from KNHANES-IV (Figure 1). 

Information about self-perceived oral health and oral functions (chewing and speaking) were collected by trained interviewers. OHS included examination of untreated decayed teeth (DT), DMFT, and prosthetic and periodontal status by trained dentists.

### 2.2. Variables

Prosthetic status was described using a six-level categorical scale (non-prosthesis = 0, one bridge = 1, two or more bridges = 2, only partial dentures = 3, bridge and partial dentures = 4, and complete denture = 5) and was determined from data of the oral examination of the upper and the lower jaws. The prosthetic status scores of the upper and lower jaws were summed (minimum score = 0 and maximum score = 10) for analysis. The community periodontal index (CPI) of examined sextants in both jaws was described using a five-level categorical scale determined by oral examination (healthy periodontal conditions = 0, gingival bleeding on probing = 1, calculus and bleeding = 2, shallow periodontal pocket 4–5 mm = 3, and periodontal pocket ≥ 6 mm = 4). The periodontal score was calculated as the mean CPI of sextants with two or more teeth (minimum score = 0 and maximum score = 4).

The self-perceived OHS score was categorized from “very good” to “very poor” (very good = 1, good = 2, fair = 3, poor = 4, very poor = 5). The perceived chewing and speaking ability score was categorized from “very comfortable” to “very uncomfortable” (very comfortable = 1, comfortable = 2, fair = 3, uncomfortable = 4, very uncomfortable = 5). The combined score of self-perceived OHS and chewing and speaking ability was estimated individually as the sum of self-perceived OHS and subjective chewing and speaking ability scores. Therefore, the combined scores of the subjective perceived OHS and functions ranged from a minimum of 3 to a maximum of 15. 

Household income level was categorized as low, middle, and high, and was adjusted for family size [21,22]. It was determined as the gross household income, divided by the square root of the number of household members. Educational level was categorized as junior high school, high school, and college and more.

### 2.3. Statistical Analysis

Statistical analyses were performed with the Statistical Package for the Social Sciences (SPSS), version 22 (IBM SPSS Statistics for Windows, Armonk, NY, USA). A plan file was produced by calculating stratified variables with a layer for dispersion estimation, clustered enumeration districts, and weighted samples with an integral weight of 3 years of existing examination survey–nutrition relation weight. The chi-squared test was used for assessing the distributions of subjects’ self-perceived oral health and subjective oral function scores with demographic socioeconomic variables. The relationship between clinical OHS and scores of self-perceived OHS and functions were analysed using a complex samples general linear model. The relationship of scores of self-perceived oral health and oral functions with chewing and speaking were analysed by age group using a complex general linear model. The coefficient values were estimated using complex samples general linear models with adjustment for related variables (estimation coefficient, B). A stepwise approach was implemented using sets of variables. Model 1 included DT, DMFT, prosthesis scores, and mean periodontal scores. Model 2 included Model 1 and demographic indicators, such as age and sex. Model 3 included Model 2 and socioeconomic indicators, such as household income and education. The level of significance was set at *p* < 0.05. 

### 2.4. Ethics Approval and Consent to Participate

Ethical approval for this study was obtained from KCDCP’s Institution Review Board (IRB No. 2007-02CON-04-P, 2008-04EXP-01-C, 2009-01CON-03-2C), and written consent was obtained from the participants by the trained investigators. 

## 3. Results

Of the subjects, 50.9% were men and 49.1% were women (Table 1). There was classification according to age group of the subjects: 51.2%, men; 48.8%, women in the aged 35 to 44 years; 50.6%, men; 49.4%, women in the aged 45 to 54 years. Table 2 shows the association between demographic socioeconomic status and self-perceived OHS of subjects, including oral functions. Self-perceived OHS differed significantly by sex, but oral functionality did not. Among subjects aged 35–54 years, 39.8% perceived themselves as having a “poor” OHS, but more than 50% were “comfortable or very comfortable” with their chewing and speaking abilities. The self-perceived OHS and functionality decreased as the subjects’ educational level decreased. Subjects of lower household income level perceived their OHS as “very poor” and “very uncomfortable” in terms of chewing ability.

The scores of the self-perceived OHS and functionality were related to age, DT, DMFT, and prosthetic and periodontal status. DT, DMFT, and prosthetic status and periodontal status scores were increased in those with self-perceived OHS or oral functions of “very poor” or “very uncomfortable” respectively (Table 3, *p* < 0.001).

The self-perceived OHS among participants aged 45–54 years was better than that among the younger group; however, there were more respondents among the former subjects who were “uncomfortable” or “very uncomfortable” with their self-perceived oral functions. The combined scores of subjective OHS and functions among subjects aged 45–54 years was higher than among subjects aged 35–44 years (Table 4, *p* < 0.001).

The combined score estimated based on the subjectively self-perceived OHS and chewing and speaking ability was associated with the clinically determined OHS. Complex samples general linear model analysis was performed to identify variables associated with self-perceived OHS and subjective oral functions. In Model 1, the self-perceived OHS and subjective oral functions were significantly associated with DT, DMFT, prosthesis scores, and mean periodontal scores (*R*^2^ = 0.127, *p* < 0.001). Increased DT, DMFT, prosthesis scores, and mean periodontal scores affected individuals’ perception of “very poor” or “poor” self-perceived OHS and oral functions. Model 2 was adjusted for sex and age (*R*^2^ = 0.136, *p* < 0.001). Increased age, DT, DMFT, prosthesis scores, and mean periodontal scores affected “very poor” or “poor” self-perceived OHS and oral functions, but sex did not. When Model 3 was adjusted for demographic and socioeconomic status (i.e., age, sex, household income level, education level), low levels of household income and education were found to be associated with worse OHS and functions (*R*^2^ = 0.148, *p* < 0.001). Clinically determined OHS was associated with self-perceived OHS and subjective oral functions in both unadjusted and adjusted models. Based on the estimation coefficient, the variable with the strongest association with self-perceived OHS and subjective oral functions was periodontal status, prosthetic status, the number of untreated decayed teeth, and DMFT, in that order (Table 5).

## 4. Discussion

In epidemiological studies, self-rated oral health is a useful summarizing indicator of OHS [23]. The present study investigated the association of self-perceived OHS and functions with clinically determined OHS. In half of the Korean adults, OHS was poor or very poor. Subjects with poor or very poor self-rated OHS tended to regard oral health less seriously than did subjects with a self-rated very good or good OHS [24]. Accurate perception of self-OHS can lead to proper oral health behaviour [25]. There were more subjects who responded with “poor” or “very poor” OHS in the 45–54-year-old age range than in the 35–44-year-old age range. As dental caries and periodontal disease are cumulative diseases, the prevalence of chewing difficulties is perceived to be lower with age [26].

One of four subjects had difficulties in chewing. Food is ingested mostly through masticatory movement of the teeth and swallowing, which is necessary for typical and efficient nutrient intake in humans [27]. Two-fold differences in poor self-rated oral health were found in elderly individuals who reported poor chewing ability and poor dental appearance. Ratios were higher in women who had never visited a dentist and in men with severe periodontal disease [12]. There were no significant differences in the chewing function between the sexes, but there were more respondents among the subjects aged 45–54 years who were “uncomfortable” or “very uncomfortable” in chewing, than among the subjects aged 35–44 years. 

It has been reported that the perception of general health and epidemiological indicators of OHS were also significant factors in perceived natural dentition status [28]. Likewise, the subjective OHS among elderly individuals in LA county was related to the objective OHS determined by a dentist [15]. Moreover, Matthias et al. explained that the number of teeth present and the number of teeth missing in the OHS as determined by a dentist were related to subjective OHS [14]. Based on the estimation coefficient of demographic and socioeconomic variables, and clinical OHS for self-perceived OHS and oral functionality, the variables with the strongest influence on self-perceived OHS and chewing ability were periodontal status, prosthetic status, and the number of untreated decayed teeth, in that order. Therefore, we considered that the self-perceived OHS and chewing ability were covariables. Our study suggested that self-reported oral health was closely related to objectively determined clinical oral health. If the subjects felt more “uncomfortable” or “very uncomfortable,” the self-perceived oral health and function score was high. The self-perceived OHS and function score increased with the higher age, DT, DMFT index, periodontal score, and prosthetic score; thus, these scores can correctly reflect the individual’s OHS.

Our findings also showed an association between demographic and socioeconomic variables and the self-perceived oral health in adults. The DT number, DMFT index, and prosthetic status and periodontal status scores increased when the self-perceived OHS or oral functions were “very poor” or “very uncomfortable”. The clinically determined OHS and age were significant influential factors in self-perceived OHS and subjective oral functions in the unadjusted and adjusted models. Although the explanatory power tended to be low, our findings suggest that self-rated OHS and oral functions are closely related to clinically determined OHS. The explanatory power of model 3 appeared to be influenced by socioeconomic variables, such as education level and income level. A poor oral health and oral status were reported to be significantly higher in adults in the lower income quintiles than in those in the highest income level. This finding is consistent with previous studies that showed that OHS was strongly associated with socioeconomic conditions [29,30,31]. Various factors have been associated with subjective oral health [12]. Age, household income, dentures, the number of lost teeth, self-esteem, quality of life, stress level, sense of belonging, and level of depression are reportedly related to subjective OHS [32]. Blizniuk et al. [33] reported that decayed teeth, missing teeth, papillary bleeding index, deep pocket depth, and the number of teeth present were factors associated with the perception oral status in 18–60-year-old adults in Belarus.

Oral symptoms that were reportedly associated with subjective OHS are uncomfortable mastication, periodontal status, and toothache; among these, uncomfortable mastication was most strongly associated with a poor subjective OHS [34]. Oral health behaviours exerted a significantly independent influence on subjective OHS and the DMFT index [35]. Previous studies, however, did not identify a relationship between self-rated OHS and clinically determined periodontal status. Kim and Lee [17] reported a strong relationship between objective and perceived OHS. When subjects feel that their oral health condition is “healthy” or “good,” the proportion of objectively determined “healthy teeth” is higher. In contrast, if individuals feel that their oral health condition is “poor”, the proportions of “filled teeth” or “missing teeth” are also high.

This study had some limitations as it was based solely on information gained from KNHANES-IV. Although we showed that the self-perceived survey on oral health and oral functions can be utilized instead of the clinical oral health survey by dentists when planning community oral health promotion programmes, we could not analyse all demographic, socioeconomic, general health, and nutritional variables that are related to subjective and objective OHS. Demographic socioeconomic variables that may be associated with subjective OHS should be included in a study of self-perceived OHS. Furthermore, it is necessary to study variables that may be associated with self-perceived general health as well as nutritional status.

Various studies have proven self-rating of oral health to be a valid and reliable method for assessing OHS [29,30,31,32,33,34], which is considerably less expensive than a clinical examination. The current study indicated that Korean adults were able to assess their OHS accurately. It could be useful for population-based oral health assessment among the civilian population of South Korea. Self-perceived OHS, based on a combination score of self-perceived OHS, was found to be reasonably valid when compared with clinically determined OHS in this study. In addition, our results indicate that it is necessary to utilize the results of a subjective questionnaire survey during the planning stage of oral health promotion programmes.

## 5. Conclusions

In adults aged 35–54 years, with similar dentition, selected from among the subjects of KNHANES–IV, self-perceived OHS based on a combined score was found to correspond fairly well with clinically determined OHS. Self-reported oral health was thus closely associated with clinical oral health. The self-rated survey of oral health and oral functions can be utilized instead of a clinical oral health survey by dentists when planning community oral health promotion programmes. Therefore, the demographic and socioeconomic variables, which may be associated with subjective OHS, should be included in studies of self-perceived OHS.

## Figures and Tables

**Table 1:** Number of subjects according to sex.

Age Group (Years)	Total	Men		Women	
		*N*	Estimate %	*N*	Estimate %
All	6605	2828	50.9	3777	49.1
35–44	3467	1487	51.2	1980	48.8
45–54	3138	1341	50.6	1797	49.4

**Table 2:** Self-perceived oral health and subjective oral function scores of subjects aged 35–54 years according to demographic socioeconomic variables.

Variables	Very Good/Very CFT ^†^	Good/CFT ^†^	Fair	Poor/UnCFT ^‡^	Very Poor/Very UnCFT ^‡^	*p*-Value *
Self-perceived oral health						
Sex						<0.001
Men	1.2	12.2	37.9	39.2	9.6	
Women	0.5	10.8	41.7	40.3	6.7	
Age group (years)						<0.001
35–44	0.7	10.7	42.5	38.7	7.4	
45–54	1.0	12.3	36.8	40.8	9.1	
Education						<0.001
≤Junior high school	0.8	10.5	35.3	42.4	11.1	
High school	0.8	10.2	41.9	39.4	7.8	
≥College	1.0	14.0	40.2	38.2	6.7	
Household income ^§^						0.003
Low	1.6	10.5	36.1	41.4	10.4	
Middle	0.7	10.9	39.4	40.0	9.1	
High	0.8	12.7	41.2	38.9	6.4	
Chewing ability						
Sex						0.142
Men	31.4	24.1	17.8	21.5	5.3	
Women	33.0	25.5	17.6	19.4	4.5	
Age group (years)						<0.001
35–44	35.1	27.5	18.0	16.8	2.6	
45–54	29.0	21.9	17.3	24.5	7.4	
Education						<0.001
≤Junior high school	27.0	18.6	16.3	27.7	10.4	
High school	33.1	25.3	18.3	19.8	3.5	
≥College	34.6	28.5	17.9	16.2	2.8	
Household income ^§^						<0.001
Low	28.9	18.5	19.1	23.6	10.0	
Middle	31.2	23.0	18.3	22.1	5.4	
High	34.4	29.0	16.4	17.2	3.0	
Speaking ability						
Sex						0.116
Men	64.6	21.6	7.9	4.9	1.0	
Women	66.4	19.3	7.5	6.0	0.9	
Age group (years)						<0.001
35–44	70.9	18.8	6.0	3.8	0.5	
45–54	59.6	22.2	9.5	7.2	1.4	
Education						<0.001
≤Junior high school	54.8	23.3	10.1	9.3	2.6	
High school	66.5	19.6	7.8	5.6	0.5	
≥College	71.8	19.4	5.9	2.5	0.3	
Household income ^§^						<0.001
Low	56.5	19.5	9.6	11.4	2.9	
Middle	64.3	20.8	8.2	5.7	1.0	
High	69.3	20.1	6.5	3.8	0.4	

* By chi-squared test using complex samples crosstabs. ^†^ CFT; comfortable, ^‡^ UnCFT; uncomfortable. ^§^ Adjusted for family size: gross household income dived by the square root of the number of household members.

**Table 3:** Clinical oral health status according to scores of self-perceived oral health status and oral functions among subjects aged 35–54 years.

Variables	DT ^†^		DMFT ^‡^		Prosthesis Scores (0–10) ^§^		Periodontal Scores (0–4) ^∥^		*p*-Value *
	Mean	SE	Mean	SE	Mean	SE	Mean	SE	
Self-perceived oral health									<0.001
Very good	0.32 ^a^	0.10	2.55 ^a^	0.50	0.15 ^a^	0.07	0.93 ^a^	0.11	
Good	0.40 ^a^	0.04	3.48 ^a^	0.15	0.38 ^b^	0.04	0.88 ^a^	0.04	
Fair	0.52 ^b^	0.03	4.98 ^b^	0.09	0.43 ^b^	0.02	0.95 ^a^	0.03	
Poor	0.83 ^c^	0.04	6.16 ^c^	0.09	0.82 ^c^	0.03	1.21 ^b^	0.03	
Very poor	1.70 ^d^	0.12	7.43 ^d^	0.23	1.32 ^d^	0.09	1.63 ^c^	0.05	
Chewing ability									<0.001
Very comfortable	0.52 ^a^	0.03	4.51 ^a^	0.09	0.40 ^a^	0.02	0.97 ^a^	0.03	
Comfortable	0.60 ^a^	0.04	5.62 ^b^	0.12	0.60 ^b^	0.03	0.96 ^a^	0.03	
Fair	0.71 ^a^	0.04	5.75 ^b^	0.12	0.63 ^b^	0.04	1.07 ^b^	0.03	
Uncomfortable	0.98 ^b^	0.05	6.21 ^c^	0.13	0.96 ^c^	0.05	1.34 ^c^	0.03	
Very uncomfortable	1.65 ^c^	0.14	6.59 ^c^	0.28	1.25 ^d^	0.11	1.75 ^d^	0.08	
Speaking ability									<0.001
Very comfortable	0.64 ^a^	0.02	5.17 ^a^	0.07	0.47 ^a^	0.02	1.03 ^a^	0.02	
Comfortable	0.86 ^b^	0.05	5.80 ^b^	0.13	0.79 ^b^	0.04	1.18 ^b^	0.04	
Fair	0.81 ^b^	0.07	6.03 ^b^	0.19	1.13 ^c^	0.09	1.22 ^b^	0.05	
Uncomfortable	0.95 ^b^	0.11	6.59 ^b^	0.29	1.40 ^c^	0.12	1.39 ^c^	0.07	
Very uncomfortable	1.47 ^b^	0.27	6.55 ^b^	0.73	1.54 ^c^	0.31	1.86 ^d^	0.19	

* By complex samples general linear model. DT mean decayed teeth. DMFT mean decayed, missing, and filled teeth. ^†^ Mean number of decayed teeth in permanent dentition. ^‡^ Mean number of decayed, missing, and filled teeth in permanent dentition. ^§^ Sum of the prosthesis scores of upper and lower jaws; none = 0, one bridge = 1, two or more bridges = 2, only partial dentures = 3, bridge and partial dentures = 4, complete dentures = 5. ^∥^ Mean community periodontal index (CPI) of examined sextants in the upper and lower jaw. CPI of sextant; healthy = 0, bleeding = 1, calculus = 2, shallow pocket = 3, deep pocket = 4. ^a,b,c,d^ Statistically significant differences are denoted by different letters.

**Table 4:** Scores of self-perceived oral health and oral functions on chewing and speaking among subjects of 35–54 years.

Age Group (Years)	Self-Perceived Oral Health * (*p* ^§^ = 0.004)		Self-Perceived Oral Functions^†^				Self-Perceived Oral Health Status and Oral Functions ^‡^ (*p* ^§^ < 0.001)	
			Chewing Ability (*p* ^§^ < 0.001)		Speaking Ability (*p* ^§^ < 0.001)			
	M ^∥^	SE	M ^∥^	SE	M ^∥^	SE	M ^∥^	SE
All	3.43	0.01	2.41	0.02	1.56	0.01	7.73	0.07
35–44	3.46	0.02	2.30	0.02	1.49	0.02	7.33	0.08
45–54	3.40	0.02	2.54	0.03	1.64	0.02	8.15	0.10

* Very good = 1, Good = 2, Fair = 3, Poor = 4, Very poor = 5. ^†^ Very comfortable = 1, Comfortable = 2, Fair = 3, Uncomfortable = 4, Very uncomfortable = 5. ^‡^ Sum of self-perceived oral health status and oral functions: minimum = 3, maximum = 15. ^§^ Complex samples general linear model. ^∥^ Estimated mean; covariates appearing in the model are fixed at the following values: DT = 0.72, DMFT = 5.46, Prosthesis scores = 0.6484, Periodontal scores = 1.1004, Sex = 0.4906.

**Table 5:** The association of demographic and socioeconomic variables, and clinical oral health status of subjects aged 35–54 years with self-perceived oral health status and oral functions.

Variables	Self-Perceived Oral Health Status and Subjective Oral Functions *								
	Model 1 (*R*^2^ = 0.127)			Model 2 (*R*^2^ = 0.136) ^a^			Model 3 (*R*^2^ = 0.148) ^b^		
	B	SE	*p* ^†^	B	SE	*p* ^†^	B	SE	*p* ^†^
(intercept)	5.309	0.13	<0.001	1.684	0.51	0.001	5.038	0.66	<0.001
Age				0.086	0.01	<0.001	0.064	0.01	<0.001
Sex ^‡^				−0.048	0.13	0.701	−0.245	0.13	0.058
Household income ^§^							−0.521	0.11	<0.001
Education ^∥^							−0.492	0.10	<0.001
DT ^¶^	0.424	0.05	<0.001	0.452	0.05	<0.001	0.405	0.05	<0.001
DMFT **	0.110	0.02	<0.001	0.112	0.02	<0.001	0.126	0.02	<0.001
Prosthesis scores (0–10) ***	0.822	0.07	<0.001	0.731	0.07	<0.001	0.691	0.07	<0.001
Mean periodontal scores (0-4) ****	0.883	0.09	<0.001	0.783	0.09	<0.001	0.700	0.09	<0.001

* Sum of oral health status score (1–5) + chewing ability score (1–5) + speaking ability score (1–5); oral health status and subjective chewing/speaking ability (oral function) score: very good/very comfortable = 1, good/comfortable = 2, fair = 3, poor/uncomfortable = 4, very poor/very uncomfortable = 5. ^†^ By complex samples general linear models. ^‡^ Male = 0, Female = 1. ^§^ Low = 0, Middle = 1, High = 2. ^∥^ ≤Junior high school = 0, High school = 1, ≥College = 2. ^¶^ Mean number of decayed teeth. ** Mean number of decayed, missing, and filled teeth. *** Sum of the prosthesis scores of upper and lower jaws: none = 0, one bridge = 1, two or more bridges = 2, only partial dentures = 3, bridge and partial dentures = 4, complete dentures = 5. **** Mean community periodontal index (CPI) of examined sextants at upper and lower jaw; CPI of sextant: healthy = 0, bleeding = 1, calculus = 2, shallow pocket = 3, deep pocket = 4. ^a,b^ Model 2, Model 3 include self-perceived oral health status and subjective oral functions adjusted by demographic variables, respectively.
